# Genetic Diversity within a Global Panel of Durum Wheat *(Triticum durum)* Landraces and Modern Germplasm Reveals the History of Alleles Exchange

**DOI:** 10.3389/fpls.2017.01277

**Published:** 2017-07-18

**Authors:** Hafssa Kabbaj, Amadou T. Sall, Ayed Al-Abdallat, Mulatu Geleta, Ahmed Amri, Abdelkarim Filali-Maltouf, Bouchra Belkadi, Rodomiro Ortiz, Filippo M. Bassi

**Affiliations:** ^1^International Center for Agricultural Research in the Dry Areas Rabat, Morocco; ^2^Department of Plant Science, Mohammed V University Rabat, Morocco; ^3^Department of Horticulture and Crop Science, Faculty of Agriculture, The University of Jordan Amman Amman, Jordan; ^4^Plant Breeding, Swedish University of Agricultural Sciences Alnarp, Sweden

**Keywords:** center of diversity, couscous, domestication, evolution, pasta, Axiom 35K, array, durum wheat

## Abstract

Durum wheat is the 10th most important crop in the world, and its use traces back to the origin of agriculture. Unfortunately, in the last century only part of the genetic diversity available for this species has been captured in modern varieties through breeding. Here, the population structure and genetic diversity shared among elites and landraces collected from 32 countries was investigated. A total of 370 entries were genotyped with Axiom 35K array to identify 8,173 segregating single nucleotide polymorphisms (SNPs). Of these, 500 were selected as highly informative with a PIC value above 0.32 and used to test population structure via DAPC, STRUCTURE, and neighbor joining tree. A total of 10 sub-populations could be identified, six constituted by modern germplasm and four by landraces of different geographical origin. Interestingly, genomic comparison among groups indicated that Middle East and Ethiopia had the lowest level of allelic diversity, while breeding programs and landraces collected outside these regions were the richest in rare alleles. Further, phylogenetic analysis among landraces indicated that Ethiopia might represent a second center of origin of durum wheat, rather than a second domestication site as previously believed. Together, the analyses carried here provide a global picture of the available genetic diversity for this crop and shall guide its targeted use by breeders.

## Introduction

Durum wheat (*Triticum turgidum* ssp. *durum* Desf., 2*n* = 4*x* = 28, AABB) is the 10th most important crop worldwide owing to its annual production of 37 million tons (LMC International, 2009; Ranieri, 2015; Taylor and Koo, 2015). It is grown on about 10% of the world’s wheat area mostly in West Asia, North, and East Africa, the North American Great Plains, India, Eastern and Mediterranean Europe (Cantrell, 1987; International Wheat Council, 1991). With the exception of Europe, North Africa (Algeria, Morocco, Tunisia, and Libya) is the largest import market for durum wheat (Bonjean et al., 2016). Its final uses vary between industrial production of pasta, couscous, and other semolina products and traditional handmade foods such as *frike*, bourghul, and unleavened breads. The vast array of homemade foods derived from durum grains is the result of its long history as part of human diets, which dates back to the origin of civilization in the Fertile Crescent (MacKey, 2005). Tetraploid wheat domestication took place about 12,000 years ago in the Fertile Crescent, when ancient farmers selected among cultivated forms of wild emmer (*Triticum turgidum* ssp. *dicoccoides*) a naked type that was easier to thresh (*Triticum turgidum* ssp. *dicoccum*; MacKey, 2005; Tanno and Willcox, 2006; Zohary et al., 2012). Approximately 2,000 years after this event, human migration and the spread of agriculture from the Fertile Crescent to and throughout Europe and Asia led to the expansion of the cultivation of naked emmer. During the same period, durum wheat (*Triticum turgidum* ssp. *durum*) appeared in the Fertile Crescent as result of further selection and domestication of naked emmer (Zohary et al., 2012). Due to its larger grains and higher productivity, durum gradually replaced its ancestor to become by the second millennium BC the major cultivated form of tetraploid wheat (Maier, 1996; Nesbitt and Samuel, 1998; Zohary et al., 2012).

Thus, durum wheat origin is the result of two successful domestication events by ancient farmers, first from wild emmer to domesticated emmer, and second from cultivated naked forms of emmer to durum (Gioia et al., 2015). The Levantine (Jordan, Lebanon, Israel, Palestine, and Syria) is considered to be the center of origin of this crop (Vavilov, 1951; Feldman, 2001). From there, it spread throughout the Mediterranean basin, probably via trading by Phoenician merchants, by the caravans’ routes along the Sahara desert or the North African coasts (Bozzini, 1988), and the Silk Road to Asia (Waugh, 2010). Reports (Mengistu et al., 2015, 2016) suggested, that durum wheat was also domesticated a third time to derive *Triticum aethiopicum* Jakubz. (syn. *Tritium durum* subsp. *abyssinicum* Vavilov), which is mainly found today under cultivation in Ethiopia and neighboring countries. It remains yet unclear if this additional domestication was the result of further modification by farmers’ of a durum landrace population originated in the Levantine, or rather if it represented a novel origin of durum by a separate domestication of naked emmer. What is clear is that the *abyssinicum* subspecies is morphologically very different, with uncompact spikes and small dark seeds (Sakamoto and Fukui, 1972; Porceddu et al., 1973; Pecetti et al., 1992; Mengistu et al., 2015).

The history of the durum wheat genetic makeup became more complex at the beginning of the 20th century when breeders started imposing artificial hybridization and selection pressure for commercial purposes (Autrique et al., 1996; Pecetti and Annicchiarico, 1998). In 1910, Nazareno Strampelli set up the first durum wheat breeding program in Foggia, southern Italy. This program was initially based on the selection of pure lines from local landraces (Scarascia Mugnozza, 2005). Later, Strampelli recognized the great value of the inheritance laws described by Mendel and started a true hybridization program. The most successful result was the cultivar ‘Cappelli,’ released in 1915 (Laidò et al., 2013). This pioneer cultivar had a major global impact in the years that followed, and most of the modern varieties can be traced back to the ‘Capelli’ lineage.

A second major impact was provided by the shuttle breeding system developed by the Nobel laureate Norman E. Borlaug several years later in Mexico and his deployment of dwarfing genes to increase harvest index (Gale and Youssefian, 1985). This resulted in the release of several semi-dwarf and widely adapted cultivars that are still grown nowadays (Ortiz et al., 2007). The modern scenario of the pedigrees post Green Revolution is extremely hard to describe, with several hybridization occurring between different breeding programs and mega-cultivars that have crossed the boundaries of their country of origin. To disentangle the last 40 years of germplasm exchange and cross hybridizations, new methods have been devised based on the allelic similarities described by molecular markers (Pritchard and Rosenberg, 1999; Christiansen et al., 2002; Falush et al., 2003; Flint-Garcia et al., 2003). A bi-product of these type of studies is the understanding of how much of the overall available alleles (namely: genetic diversity) have been captured within a specific germplasm. Since genetic diversity is often seen as an essential source of novel and useful alleles to be selected by breeders (Tanksley and McCouch, 1997; Cooper et al., 2001; Spillane and Gepts, 2001; Acosta-Gallegos et al., 2007), these types of studies have both a historical value and an immediate practical impact on breeding. Hence, the aim of this research was to conduct a molecular assessment of a global durum wheat collection of cultivars, elite breeding lines and landraces, in order to photograph the current state of germplasm exchange and overall available genetic diversity.

## Materials and Methods

### Plant Material

A large durum wheat germplasm collection exceeding 1,500 accessions was assembled at the field station of the International Center for Agricultural Research in the Dry Areas (ICARDA) in Terbol, Lebanon (33° 49′ 05″ N, 35° 58′ 59″ E). A core subset was defined after assessing the collection for similarity in flowering time, response to toxic level of boron, disease response, tendency to lodge, visual selection, and characterized with 10 single nucleotide polymorphisms (SNPs) associated to known genes. The original set contained several landraces selected on the basis of the algorithm for Focus Identification of Germplasm Sources (FIGS; Mackay et al., 2005; Bari et al., 2012) targeting the model to identify sources of rust resistance, tolerance to drought, heat and mineral toxicity. A core subset of 384 accessions was selected to be similar in phenology and diverse for all other traits. It includes 96 landraces from 24 countries and 288 cultivars and elite breeding lines from eight countries, ICARDA, and International Maize and Wheat Improvement Center (CIMMYT). This panel was built on the work already carried on by Maccaferri et al. (2003), removing duplicates and adding a set of landraces, new breeding material from ICARDA, CIMMYT, Canada, and Australia (**Supplementary Figure S1**). A detailed list of materials is provided in **Supplementary Table S1**.

### DNA Extraction and Genotyping

DNA was extracted from leaf samples using a standard cetyltrimethylammonium bromide (CTAB) protocol (Doyle and Doyle, 1987). The 384 accessions were genotyped by 35K Affymetrix Axiom wheat breeders array^1^ at Trait Genetics (Gatersleben, Germany) following the manufacturer instructions. This array was developed by choosing tags of proven high polymorphism when tested on modern bread wheat elites, among the 817k SNP Axiom HD platform.

### Data Analysis

The polymorphic information content (PIC) was calculated following the formula described by Botstein et al. (1980), and the 2-points LOD was generated using Carthagene software option ‘SEM’ (Schiex et al., 2009). The discriminant analysis of principal components (DAPC), was performed using the ‘adegenet’ package 1.4-1 (Jombart et al., 2010) in R studio V 2.3.2 (R Development Core Team, 2011). With DAPC, the genetic variation was decomposed using a multivariate ANOVA model as:

Total variance VAR(X) =variance between groups B(X)+variance within groups W(X)

**FIGURE 1:**
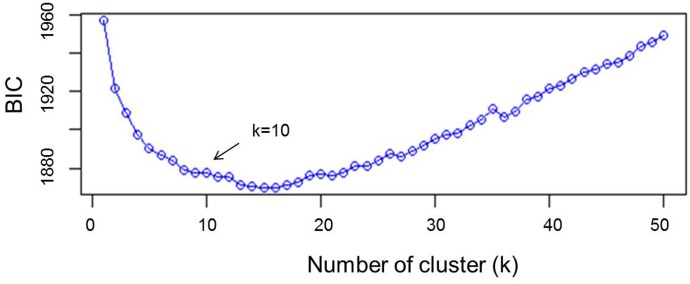
Statistical determination of the optimum number of clusters by discriminant analysis of principal components (DAPC).

Other approaches such as principal component analysis (PCA) or principal coordinates analysis (PCoA/MDS) focus on VAR(X). That is, they only describe the global diversity, possibly overlooking differences between groups (Jombart and Collins, 2015). The variance explained by PCA was fixed to 75 and the value of *k* was tested from 2 to 50. The rate of decrease of the Bayesian information criterion (BIC) was visually examined (**Figure 1**), and the number of clusters was determined as the value of *k* above which BIC values decreased. Analysis of admixture by kinship was performed using the Bayesian clustering algorithm implemented in the software STRUCTURE v 2.3.4 (Pritchard et al., 2000) using 50,000 burning periods and 10,000 replicates and re-assessed five times with 11 independent runs. The value of *k* was set based on DAPC results. To further confirm cluster analysis, unweighted pair group method of association (UPGMA) was carried out using the genetic similarity matrix by numerical taxonomy and multivariate analysis system (NTSYS-PC) version 2.02e software (Rohlf, 1997). Because this method uses genetic similarity matrix, a line of reference was arbitrary set to explain 60% of similarity in order to determine the genetically distinct branches of the tree. Arlequin 3.5.2.2 (Excoffier et al., 2005) was used to assess the molecular variance (AMOVA) between clusters. Phylogenetic studies of landraces were conducted by neighbor-joining algorithm of the genetic distances determined by STRUCTURE using 1,000 bootstrapping analysis for an unrooted tree by DARwin V 6.0.12 software (Perrier et al., 2003). DIVA-GIS V 7.5.0 software (Hijmans et al., 2012) was used to graphically map the GPS coordinates of the places of collection of the landraces.

## Results

### Genotyping of a Global Panel of Durum Elites, Cultivars, and Landraces

A total of 384 durum entries were genotyped, but only 370 showed DNA quality sufficient for SNP calls. In total, 35,143 SNPs were assessed, of these 11,642 (34%) failed to meet the minimum call rate, which suggests that these markers were probably located on the D genome, present in hexaploid bread wheat but not in tetraploid durum wheat. A total of 14,851 (42%) met the quality cutoff but remained monomorphic in this population, while 8,173 (36%) were found to be high quality and polymorphic. The average frequency of the minor allele was 12% with a minimum of 3%. **Table 1** provides the chromosome assignment of markers based on the work by Winfield et al. (2016) in bread wheat, where 1,559 markers remained unassigned.

**Table 1:** Number and distribution across the 14 chromosomes (Chr.) of durum wheat of polymorphic SNPs markers on the Axiom 35K breeder’s array and the 2-points LOD for the subset of the 500 SNPs used for clustering.

			2-points LOD of the subset		
			
Chr.^a^	Polymorphic	Subset of 500 SNPs	Average	MIN	MAX
1A	505	26	36.7	0.3	101.2
1B	617	48	33.9	0.0	111.1
2A	519	31	36.8	0.0	111.1
2B	589	28	23.7	0.0	110.8
3A	411	22	36.8	0.0	110.5
3B	533	41	30.8	0.1	111.4
4A	306	25	35.8	0.0	101.5
4B	283	22	39.6	0.0	110.8
5A	489	33	35.7	0.0	110.2
5B	673	48	31.4	0.0	110.2
6A	360	22	49.4	0.0	111.1
6B	480	40	26.1	0.0	111.1
7A	505	35	36.5	0.0	110.5
7B	344	41	41.4	0.0	111.4
Unassigned	1,559	38	8.5	0.0	78.0
Total	8,173	500			


*^a^Chromosome assignment was done on the basis of a consensus bread wheat genetic map (Winfield et al., 2016).*

### Population Stratification

**FIGURE 2:**
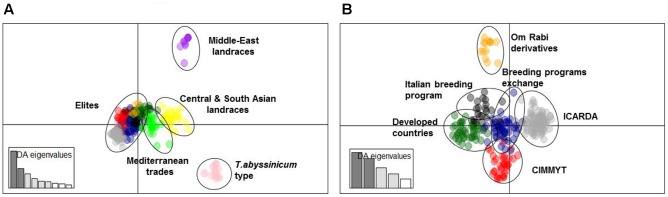
Cluster analysis using DAPC. **(A)** Graphical representation of principal component 1 (IPC1) and 2 (IPC2) distances for 10 sub-populations within the whole panel. **(B)** Graphical representation of IPC1 and IPC2 distances for 6 sub-populations among modern germplasm.

**FIGURE 3:**
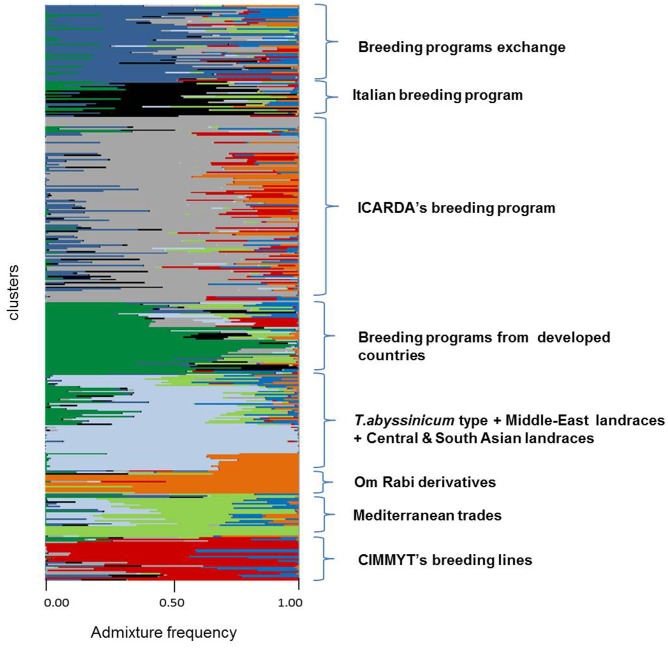
Admixture analysis by kinship of 370 durum wheat landraces and modern elites. Each individual is represented by a horizontal line. Color codes follow the color assignment of **Figure 2**, with the exception of clusters 1 to 4 that were merged into one color (one cluster) when tested by STRUCTURE (light blue).

A subset of 500 highly polymorphic (0.32 ≤ PIC ≥ 0.45) markers was chosen for clustering and kinship studies. These markers were selected for even distribution across the genome, covering all durum chromosomes, with LOD values that ranged from 0 to 111.1, and averaged at a minimum of 23.7 in chromosome 2B (**Table 1**). These LOD scores indicate good distribution and correct chromosome assignment. DAPC inferred the optimum number of sub-populations to be 10 (**Figure 1**). AMOVA was used to determine that variation among and within groups was highly significant (*P* < 0.001), with the clusters capturing 31.5% of the total genetic variations, while 68.3% was explained by individuals within populations (**Table 2**). Among the 10 clusters, four groups were composed of landraces, while six groups included mostly cultivars and elite lines (**Figure 2**). STRUCTURE was also used to determine cluster assignment, with the strongest contradiction between DAPC and STRUCTURE identified among landraces. In fact, only two clusters were identified by the latter compared to the four by DAPC (**Figure 3**). This issue was circumvented by running separately landraces and modern lines in STRUCTURE, in which case good agreement could be found between the two software. Instead, tree-based studies by UPGMA identified six clusters determined at 60% of similarities, three composed of mostly landraces and three by modern lines. This value was kept as identified. Entries were assigned to cluster based on DAPC study as it was considered the most reliable method, but a qualitative score was given to each assignment as ‘solid’ when the three methods agreed in the assignment, ‘good’ when two methods agreed, and ‘bad’ when only DAPC made the assignment. Among 370 lines, 16 (4.3%) were scored as ‘bad,’ 70 (18.9%) as ‘good,’ and 284 (76.7%) as ‘solid’ (**Table 3**). In particular, sub-populations 3 and 6 had the highest number of ‘bad’ assigned entries. Clusters 1, 2, 5, 8, and 9 were the most solid with no ‘bad’ assignments, and few ‘good.’ Full details for each genotype are provided in **Supplementary Table S1**.

**Table 2:** Analysis of molecular variance (AMOVA) for stratification of a global durum wheat panel into 10 sub-populations.

SOV	d.f.	Sum of squares	Variation (%)
Populations (P)	9	20,906^∗∗∗^	0.315
Individuals within P	360	49,809^∗∗∗^	0.683
Individuals	370	67^∗∗∗^	0.002
Total	739	70783	


*^∗∗∗^Indicate that source of variation was highly significant at *P* ≤ 0.001.*

**Table 3:** Reliability of the entries assignment based on comparison between three genetic clustering methods: DAPC, STRUCTURE, and neighbor-joining (details available in **Supplementary Table S1**).

		Reliability score		
		
DAPC Cluster IDs	Assigned entries (*N*)	Bad	Good	Solid
1. Middle East	11	0	0	11
2. *T. abyssinicum* type	18	0	0	18
3. Mediterranean trades	26	7	8	11
4. Central and South Asian	27	1	4	22
5. ‘Om Rabi’ derivatives	13	0	1	12
6. Italian cultivars	26	6	5	15
7. Breeding program exchange	58	2	29	27
8. Developed countries	30	0	4	26
9. ICARDA derived	119	0	5	114
10. CIMMYT derived	42	0	14	28
Total	370	16	70	284


Cluster 1 comprises 11 landraces from West Asia (Levantine). Cluster 2 is represented by 18 landraces, 15 from Ethiopia, 1 from Yemen, 1 from Jordan, and 1 from Russia. Cluster 3 is composed of 26 landraces, 5 from Tunisia, 4 from Algeria and Spain, 2 from Afghanistan, Greece, and Italy, and 1 each from Azerbaijan, China, Ethiopia, Iran, Kazakhstan, and Russia. Cluster 4 is composed mainly of Central and South Asian landraces and includes those collected in Afghanistan, Armenia, Georgia, India, Iran, Iraq, Kazakhstan, Pakistan, Turkey, Russia, but also Italy, Oman, Yemen, and Saudi Arabia. Cluster 5 gathers 13 modern lines from the breeding program of ICARDA, which include in their pedigree ‘Om Rabi’ – a line derived from the cross between the elite ‘Jori’ and the Jordanian landrace ‘Haurani,’ one Italian landrace and the Italian cultivars ‘Arcangelo,’ ‘Appio,’ and ‘Capeiti.’ Cluster 6 contains 20 modern lines and 6 landraces, with 13 cultivars and 2 landraces from Italy, 4 landraces from Ethiopia, and the remaining modern germplasm from France, ICARDA and Spain. Cluster 7 is represented by 58 entries from different breeding programs, including 24 elites from ICARDA, 6 from CIMMYT, 4 cultivars from France and USA, 7 from Italy, 6 from Morocco and Spain, but also 1 landrace each from Spain and France. Cluster 8 includes 17 cultivars from North America, 5 from Australia, 2 from France, 1 from Italy and Spain, plus 2 landraces from Algeria and 1 landrace selection (‘Shabha’) from ICARDA. Cluster 9 is the largest with 106 breeding lines from ICARDA, 2 from CIMMYT, 4 varieties from Italy, 4 from Morocco and Tunisia, ‘Wallaroi’ from Australia, and 2 Moroccan landraces. Cluster 10 groups 24 elite lines derived from the breeding program of CIMMYT, 1 from ICARDA, 9 Australian cultivars, 5 Spanish, 2 Moroccan, and 1 Iranian landrace. The clustering of the panel is presented in **Figure 2**, and modern lines are detailed in **Figure 2B**.

### Admixture Analysis by Kinship

**FIGURE 4:**
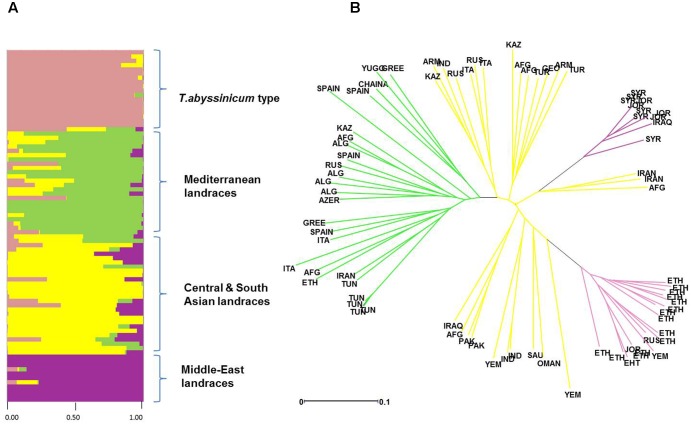
Diversity in admixture among landraces by *ad hoc* STRUCTURE analysis. **(A)** Admixture analysis by kinship color coded following the colors of **Figure 2**. **(B)** Phylogenetic tree of evolutionary distances based on kinship values color coded following the colors set in **Figure 2**.

**FIGURE 5:**
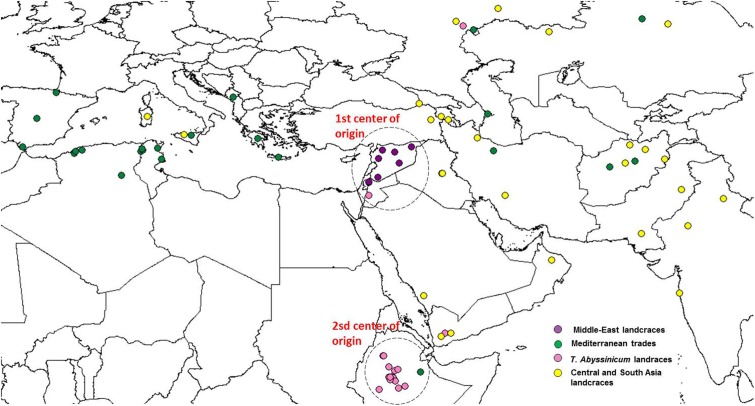
Geographical distribution of the coordinate of collection of the durum wheat landraces. Color codes are provided following the colors set in **Figure 2**. Dashed circles indicate the two centers of origin or diversity.

Admixture analysis was conducted using STRUCTURE. To better detail the kinship among landraces, these were also analyzed alone in the form of a phylogenetic analysis (**Figure 4**). Four main branches could be identified as defined by clusters 1, 2, 3, and a part of 4. In fact, cluster 4 containing mostly Central and South Asian landraces had the highest level of admixture, it located toward the origin of the tree, and created one independent branch with seven landraces from Armenia, Kazakhstan, India, Russia, Afghanistan, Turkey, and Georgia. Still, landraces from the same cluster also distributed to the branches generated by cluster 2 and 3. Cluster 2 containing mostly landraces from Ethiopia generated the branch further away from the origin of the tree, with landraces of cluster 4 from Iraq, Afghanistan, India, Pakistan, Yemen, Saudi Arabia, and Oman that located along this branch (**Figure 4**). Cluster 1 generated an independent phylogenetic path, with landraces from Syria, Jordan, and Iraq composing the edge of the branch. Cluster 3 included landraces from many countries. The coordinates of the collection sites of the landraces were placed on a map and color coded to match their cluster assignment (**Figure 5**). The admixture level among elites was higher than for the landraces (**Figure 3**). Cluster 9 containing ICARDA breeding lines was the largest group, and it is therefore unsurprising that it also presents the highest level of admixture, followed by cluster 7 of *breeding exchange*. Cluster 6 of the *Italian breeding program* has noticeable allelic similarity with cluster 3 of *Mediterranean trades*, but also with cluster 8 of breeding programs from *developed countries*. The cluster of *Om Rabi derivatives* is the smallest group, which shows a low level of admixture, but still it has some alleles in common with the cluster of ICARDA breeding program. Also the CIMMYT’s elites group shared an important amount of alleles with the latest cluster.

### Genetic Diversity among 10 Sub-populations

A second set of 500 markers capturing the rarest alleles (0.005 ≤ PIC ≥ 0.01) and the full set of 8.2K polymorphic markers were used to further assess allelic frequencies (**Table 4**). Cluster 1 presented the lowest genetic diversity with PIC = 0.04, followed by Cluster 5 (PIC = 0.05), these two clusters also had the lowest frequency of segregating markers 0.13 and 0.19, respectively, and did not capture any of the rare alleles. The group of Ethiopian landraces (cluster 2) also had a low level of segregating alleles (42%) comparable to what was observed for breeding programs from developed countries (cluster 8), and equally captured very few rare alleles (1%). The Central and South Asia landraces was the most genetically diverse sub-population, with a PIC of 0.22, almost all markers segregating (0.84) and also captured 62% of the rare alleles. This was also the cluster with the highest admixture that distributed along all other phylogenetic branches. Among the modern germplasm, clusters 7 and 9 were the most diverse, with 58 and 51% of total alleles captured, and 4 and 3% of rare alleles represented in the sub-populations, respectively. Interestingly, cluster 10 maintained the highest level of rare alleles (21%) among the modern germplasm.

**Table 4:** Genetic diversity captured by each sub-population.

		All markers			Rare alleles		
			
DAPC cluster IDs	*N*	Fixed^a^ (%)	Segregating (%)	PIC^b^	Fixed^a^ (%)	Segregating (%)	PIC
1. Middle East	11	0.77	0.13	0.04	1.00	0.00	0.005
2. *T. abyssinicum* type	18	0.47	0.42	0.13	1.00	0.01	0.005
3. Mediterranean trades	26	0.35	0.65	0.17	0.94	0.06	0.005
4. Central and South Asian	27	0.15	0.84	0.22	0.38	0.62	0.005
5. ‘Om Rabi’ derivatives	13	0.78	0.19	0.05	1.00	0.00	0.005
6. Italian cultivars	26	0.53	0.46	0.11	0.99	0.01	0.005
7. Breeding program exchange	58	0.42	0.58	0.14	0.96	0.04	0.005
8. Developed countries	30	0.56	0.43	0.12	0.99	0.01	0.005
9. ICARDA derived	119	0.48	0.51	0.11	0.97	0.03	0.005
10. CIMMYT derived	42	0.56	0.44	0.10	0.79	0.21	0.005


*^a^ Refers to major allele.*

*^b^ Polymorphism information content.*

## Discussion

Genetic diversity is of paramount importance as a source of novel traits and alleles for plant breeding, particularly to face the unpredictable challenges laying ahead, at a time of changing climates and new end-user demands (Tester and Langridge, 2010). However, diversity *per se* is of limited use (Frankel et al., 1995; Royo et al., 2009; Novoselović et al., 2016). It is instead to the breeders’ advantage to know which ideal sources of diversity should be integrated within each program to better target their crossing schemes. With this scope, the global diversity of durum wheat was assessed comparing breeding efforts, historical cultivars, and landraces from 28 countries. Genotyping with the Axiom 35K “breeders’ array” revealed that the panel used could capture 36% of total polymorphism existing for the A and B genomes markers available on the array. A subset of 500 highly informative SNP markers was used to assess the genetic structure and stratification of the panel. This number of markers was in excess as compared to what previously reported in the literature (Maccaferri et al., 2003; Raman et al., 2010; Royo et al., 2010; Cabrera et al., 2015). Overall, the genotyping results were satisfactory and allowed the implementation of all downstream applications.

### Success Level of the Clustering Procedure

Human practices such as farming, consumption habits, and trading of seeds within and among communities generate pressure bias, drift or founder effects on the germplasm (Dyer and Taylor, 2008; Delêtre et al., 2011). Furthermore, societal, cultural, and natural barriers reinforce reproductive isolation, limiting or encouraging gene flow among cultivars (Pusadee et al., 2009; Deu et al., 2013). Thus, several factors can influence the genetic diversity within a germplasm collection and the analysis presented here can only explain a fraction of it. The results of the AMOVA confirmed that the DAPC model was able to capture approximately one fifth of the total variance by stratifying the panel into 10 clusters, with individuals that maintained high levels of genetic diversity within groups. Thus, even if the choice of *k* = 10 was conservative as shown by the AMOVA, it was considered adequate to better identify similarities between genotypes, rather than over-fit their differences (Jombart et al., 2010). Clustering landraces by allelic similarities (kinship and admixture) is *de facto* an attempt of tracing those alleles that are identical by descent, hence maintained from their original domestication event or shared environmental pressures. Instead, in the case of cultivars and elite lines, genetic similarity is strongly influenced by the breeders’ subjective choice of hybridizing specific germplasm sources to develop new lines. Since different breeding programs tend to utilize the same founders in their crossing strategies, strong admixtures exist between geographically distant germplasm. Separating cultivars and elite lines into groups of shared allelic similarity is therefore an attempt to capture the complex hybridization history of the breeding germplasm.

The population stratification was done via DAPC (**Supplementary Figure S2**), STRUCTURE analysis, and neighbor joining method. While some disagreement was found among methods, only 4.3% of tested genotypes were not assigned to the same clusters by two or more of the software used for the analysis (**Table 3** and **Supplementary Table S1**). Considering the high level of admixture showed by the landrace sub-group identified by STRUCTURE, and the clustering by UPGMA tree (**Supplementary Figure S2**), the results of DAPC were deemed more reliable.

### History of Durum Origin and Migration Based on Genetic Diversity of Landraces

The population stratification of this panel identified four groups of landraces and six groups of cultivars and elite lines. Cluster 1 includes landraces from Jordan, Syria, and Iraq (**Figure 4**), countries that correspond to the center of origin of durum wheat in the Levantine (MacKey, 2005). The geographical proximity of these landraces to the center of origin maintained a high level of genetic purity with low levels of admixture (**Figure 4**) and almost complete fixing of major (77%) and minor (90%) alleles (**Table 4**). This is in good agreement with what was reported previously for landraces from Jordan (Rawashdeh et al., 2007; Mohammadi et al., 2014). More interestingly, the phylogenetic tree (**Figure 4**) clearly indicates how the germplasm from Syria and Jordan are more closely related as compared to the germplasm from Iran and Afghanistan belonging to cluster 4. This would suggest that durum wheat truly originated in the South end of the Fertile Crescent (MacKey, 2005), and only later migrated to the neighboring regions.

Cluster 2 groups mostly landraces from Ethiopia, with the exception of one from Yemen, one from Jordan, and another from Russia. However, the Russian landrace appears as wrongly assigned based on its high level of admixture (**Figure 4**). Ethiopia is known as a “secondary center of durum wheat diversity” (Harlan, 1969; Vavilov, 1992). Landraces from this country have unique morphology (Sakamoto and Fukui, 1972; Porceddu et al., 1973; Pecetti et al., 1992) and represent a separate sub-species under the name *T. durum* subs. *abyssinicum* or *T. aethiopicum* (Mengistu et al., 2015, 2016). **Figure 4** clearly shows that this germplasm is distinct from the primary region of origin of durum wheat (Middle-East landraces) with substantially no kinship to it. Furthermore, there is limited admixture between this group and any others. Hence, Ethiopia truly represents a center of diversity for durum wheat, without an evident allelic similarity to the primary origin in the Levantine, as also suggested by several other authors (Sakamoto and Fukui, 1972; Porceddu et al., 1973; Pecetti et al., 1992). The lack of allelic similarity between the two centers of diversity can be due to adoption in Ethiopia of a population of landraces from the Middle East that was genetically different from those that can be found there today (founder-migration effect), or as a separate domestication of *T. dicoccum to T. durum*. While both explanations are valid, the tight geographical distribution and low admixture observed among the landraces from the Levantine do not support the hypothesis that a population of landrace existed within this region and then migrated to Ethiopia. However, caution should be used here as one Jordanian landrace was identified among this cluster with 99% of genetic similarity to *T. abyssinicum* types. This landrace does not show the traditional relaxed spike morphology of the *T. abyssinicum* type (**Supplementary Figure S3**) so it remains extremely hard to conclude if this indeed represents the one original landrace population that migrated from the Levantine to Ethiopia. Certainly, its complete lack of genetic similarity to the other Levantine landraces seems to suggest that it was more likely migrated from Ethiopia back to the Levantine, rather than the opposite direction. The second hypothesis then appears slightly more plausible. Domesticated emmer reached Ethiopia some 5,000 years ago (National Research Council, 1996) probably arriving from Egypt along the Silk Road (Luo et al., 2007) and it occupies approximately 7% of the wheat production today under the name of *aja*. Thus, it can be suggested that Ethiopia is indeed not a secondary center of diversity, but rather a “secondary center of origin,” where emmer was further domesticated to durum wheat as it occurred in the Levantine more than 7,000 years before. More targeted study of Ethiopian emmer genetic similarities to landraces from the Levantine and Ethiopia would be need to reach a final conclusion.

Cluster 3 unifies the landraces from the Mediterranean basin (Italy, Greece, Tunisia, Algeria, and Spain), plus few originating from Ethiopia, Afghanistan, Azerbaijan, Kazakhstan, China, Yugoslavia, Iran, and Russia. Foremost, the landrace collected from Ethiopia that belong to this group do not show the typical morphology of *T. abyssinicum* (data not shown). Therefore, it is possible that even if these were collected in Ethiopia, they might not be of the *T. abyssinicum* type. A simple explanation might be that these are rather historical Italian cultivars derived from landraces (Scarascia Mugnozza, 2005) and brought to Ethiopia during the occupation by Italy from 1936 to 1941. Concerning the other countries, it is possible that these landraces are also of Italian origin and from there spread to the neighboring countries through trading in the last millennium. However, with the exclusion of the similarity between Italian and Ethiopian landraces, in the phylogeny tree all genotypes branch out directly from the origin of the branch (**Figure 4B**), which indicates the existence of strong genetic differences among individuals. In fact, this population holds high level of genetic diversity (PIC = 0.17), low levels of alleles fixation (35%), and high level of admixture. Thus, the hypothesis of one single common origin from Italy appears unlikely. In fact, their shared allelic identity suggests that these might have originated from related seed sources, which have then be exposed to similar natural pressures by the environment and accumulated distinct mutations over time.

Cluster 4 is the largest among landraces, the most genetically diverse overall, and it clusters entries from 18 countries (**Supplementary Table S1**). It also shows severe levels of admixtures to the three clusters described above (**Figure 4**). These landraces are therefore likely the result of migration and hybridization of germplasm belonging to clusters 1, 2, and 3. This cluster can be further divided into four sub-populations based on their admixture levels (**Figure 4**). The sub-groups 1 and 2 are derived primarily from Central Asia (Kazakhstan, Afghanistan, India, Armenia, Turkey, Russia, Georgia, and Italy). They are evolutionarily closer to the “Mediterranean types,” but maintain good distinction with little similarity among individuals. As described for Cluster 3, this type of similarity with strong individual diversity is probably best explained as multiple separate sampling events from a common seed source, combined with shared environmental pressures. Thus, a scenario can be devised where merchants or tribes departing the Levantine for Central Asia carried with them seeds from neighboring fields. The third sub-group includes entries collected in the Fertile Crescent (Iraq, Afghanistan, and Pakistan), South Asia (Yemen and India), and the Arabian Peninsula (Oman, Saudi Arabia, and Yemen). This group locates evolutionary along the branch of *T. abyssinicum* types. In particular, the landraces from Yemen and Oman are more closely related to the Ethiopian landraces, and are therefore the probable result of dispersion from this secondary center of origin or domestication of durum wheat. The last sub-group is composed of landraces from Iran and Afghanistan located in the same branch with Middle-East landraces, and thus likely dispersed from here.

A special note is required for the landraces of Russian origin. These were identified in clusters 2, 3, and two sub-groups of cluster 4. This level of genetic diversity is normally unexpected for a country so geographically distant from the two centers of origin of durum wheat. However, this vast region has witnessed large migration since its origin, a well-documented source of genetic variation (Vavilov, 1951, 1992).

### History of Breeding Exchange and Cross-Hybridization as Explained by the Genetic Diversity of Modern Germplasm

Eleven landraces from the core subset were grouped within the clusters of cultivars and elite lines. The simplest explanation is that these were not true landraces, but rather old tall cultivars that were wrongly labeled during the collecting missions by the gene banks. Alternatively, landraces have often been considered a key resource for contemporary agriculture and thus have been used in plant breeding programs to enlarge the genetic diversity of modern genetic pools (Bradshaw and Ramsay, 2005; Sharma et al., 2013). Hence, it is possible that these landraces are among those utilized in recent years to improve biotic and abiotic tolerance, or were favored by the early breeders like Nazareno Strampelli to develop pure lines (Scarascia Mugnozza, 2005).

Cluster 5 is small, composed of just 13 elite lines and cultivars from ICARDA breeding program and most of them include the cultivar ‘Om Rabi’ in their pedigree. Om Rabi is the name of the largest river of Morocco, and this name was attributed to one of the first cross ever produced by the ICARDA durum breeding program in 1981, which combined the widely cultivated Jordanian landrace ‘Haurani’ with the successful CIMMYT line ‘Jori69.’ Cultivars have been released in 12 countries from this cross under various names (‘Cham 5,’ ‘Tomouh,’ ‘Om Rabi,’ ‘Oum Rabi,’ ‘Omrabi,’ ‘Gahar,’ ‘Um Qais,’ and ‘Aydin93’) and they remain widely cultivated by smallholder farmers in the most dry areas of central West Asia and North Africa. Considering that 50% of the genome of this cross is derived from a Levantine landrace, it is not surprising that it shares admixture with Cluster 1, and it has very similar allelic fixation as the landraces from the center of origin of durum wheat (**Table 4**).

Cluster 6 brings together modern and old cultivars developed by Italian breeders. Substantially this set of lines is derived from the initial work of Nazareno Strampelli and the following “fathers” of Italian breeding (Scarascia Mugnozza, 2005). The admixture level is high (**Figure 3**) and it captures 46% of the total alleles assessed (**Table 4**), indicating that several breeding programs worldwide utilized the work carried on in Italy as a base of their cultivar development pipeline. However, the level of genetic diversity is low compared to other breeding clusters (PIC = 0.11), which could be the result of the frequent use in hybridization of a reduced number of founders, in combination with strong selection pressure for the same traits needed for the Italian growing conditions and the rheological requirements of the pasta industry.

Cluster 7 is located at the center of the graph of the two main IPC by DAPC (**Figure 2**) and it groups together material from several countries and breeding programs such as Spain, Morocco, ICARDA and CIMMYT. It originates from the sharing of several germplasm sources among breeders targeting similar Mediterranean growing conditions. Thus, the genetic similarity between this germplasm can be explained as the common origin of allelic sources, together with the imposition of similar selection pressure for specific traits. This cluster has the highest rate of genetic diversity (PIC = 0.14) and portion of captured alleles (58%) than any other cluster of modern germplasm. In addition, the high admixture (**Figure 3**) and central position in the DAPC graph (**Figure 2**) confirm that this cluster is the founding base that guarantees good exchange of alleles among all other breeding programs.

Germplasm from USA, Australia, and Canada were grouped together in cluster 8, together with four lines from Italy, Spain, and France and two landraces from Algeria. This cluster captures the least amount of available allelic variation (43%) or rare alleles (1%) among breeding programs, and one of the lowest PIC (0.12). Considering the geographical distance between the breeding programs grouped here, and the different environmental conditions, it is a good example of the decay in genetic diversity that other authors have suggested is occurring in breeding programs worldwide (Hoisington et al., 1999; Martos et al., 2005). The tight rheological requirements imposed by the pasta industry has pushed durum wheat breeders to maintain their hybridization programs extremely narrow, using often the same set of standard cultivars as donors of quality traits (Karagöz and Zencirci, 2005; Zencirci and Karagoz, 2005; Altintas et al., 2008). This is reflected by the high number of fixed alleles identified in this cluster (57%) for which genetic diversity no longer exist within these breeding programs. Still, it is important to also indicate that for a large portion of the genome (43%) genetic diversity was captured and can be exploited to make further genetic gain.

Cluster 9 groups together the vast majority of the germplasm of ICARDA included in this analysis, with the exclusion of the ‘Om Rabi’ derivatives assigned to Cluster 5, and some of the genotypes included in Cluster 7. The durum wheat breeding program of ICARDA officially started in 1981 and run for over 20 years under the umbrella of CIMMYT. This program primarily targeted drylands agriculture using crossing schemes involving both modern and primitive germplasm. It released over the years 100 cultivars in 25 countries (Lantican et al., 2015). Within this group are also included some of the Italian cultivars derived from ‘Creso,’ a radiation mutant of Strampelli’s cultivar ‘Capelli,’ and several of the CIMMYT lines derived from ‘Yavaros,’ a CIMMYT cultivar that spread widely in North Africa and is today the most grown in Morocco, Algeria, and Tunisia under the name of ‘Karim’ (syn. ‘Bittern’). The genetic similarity between ‘Creso,’ ICARDA’s and CIMMYT’s materials can be found in the pedigree of ICARDA’s breeding lines, which widely used ‘BiCre’ as a parent. In fact, ‘BiCre’ is derived by the simple cross of ‘Bittern’ and ‘Creso.’ This cluster captures 51% of the total allelic diversity available in the panel, and 3% of the rare alleles. Due to the large size (*N* = 119) the PIC is low. This shows that even a breeding program that specifically targets genetic diversity as an adaptation strategy via frequent crosses to primitive germplasm can erode large parts of it by exposing the germplasm to severe selection pressures in challenging environments. Still, this cluster is the second most genetically wide among modern germplasm. Also, ICARDA’s breeding lines spread over other two clusters, thus meaning that overall the program was able to maintain acceptable levels of diversity.

The breeding program of CIMMYT has been running for over 50 years. It has had the ability to deliver superior cultivars throughout the developing world and still serves today as source of useful alleles for industrialized countries. As for the ICARDA’s cluster, the severe selection pressure during breeding caused a shrinkage in the overall genetic diversity, with the vast majority of the CIMMYT’s germplasm clustering mostly in one group (Cluster 10) with the lowest PIC (0.10) and 56% of the genome in fixed status. However, this cluster also captures 21% of the available rare alleles, which is by far the best achievement in that sense among breeding programs. Furthermore, CIMMYT breeding lines can also be found in clusters 9 and 7, which suggest an overall high level of allelic diversity remains available for breeding advancements.

### Comparison to Other Population Stratification Studies of Durum Wheat

In previous research, a panel of 190 Spanish durum wheat landraces was attributed to nine sub-populations (Ruiz et al., 2012), while a similar set of the germplasm collection used here, comprising 134 modern durum cultivars, was assigned to six sub-populations by Maccaferri et al. (2003). In our research, the number of clusters used for stratification could have been increased, to allocate four additional sub-populations among landraces of Cluster 4. However, the setting of *k* is highly dependent on the scope of the research and here the preference was given to capturing similarities rather than divergences. A large portion of admixture among landraces remained unfixed with the set value of *k*, and this could justify the difference in the number of clusters between our work and that of Ruiz et al. (2012). The ability to distinguish following waves of dispersion among landraces was one of our scopes and this was achieved by finding separation between the two main centers of origin/diversity (Middle East and Ethiopia) and other landraces. Similarly, the division into six clusters of modern material appeared in line with the results of previous authors (Maccaferri et al., 2003) and it provided interesting information about the history of alleles exchange among breeding programs. Slight differences were, however, observed from past works, due to the significant increase in this study of the number of elites derived from the ICARDA breeding program, which alone defined two novel well distinct clusters (5 and 9), and also the study of recent Australian and Canadian cultivars, which also created a cluster not described before by Maccaferri et al. (2003).

### Genetic Diversity and the Future of Durum Wheat Breeding

The scenario of today’s global cultivation of durum wheat can be summarized in the work of few great breeders: North Africa and the Middle East countries still heavily rely on ‘Karim’ (syn. ‘Bittern,’ ‘Yavaros79’) a mega-cultivar bred by Dr. Gregorio Vazquez (CIMMYT), similarly ‘Simeto’ is probably the most grown cultivar around the World and it was developed in Italy by Dr. Fortunato Calcagno (Pro.Se.Me), and the ‘Cham’ series that occupy some of the driest areas of the World were bred at ICARDA in Syria by Dr. Miloudi Nachit. The scenario of the industrialized World is only slightly more segmented, with few mega-cultivars also occupying significant land area. As shown by this genetic diversity study, most of the modern germplasm has only 58 to 44% of the genes still segregating, regardless of the breeding strategy or combination of germplasm utilized. Unexpectedly, the two centers of origin of durum wheat do not appear to be the most exploitable source of allelic diversity with most of their loci in fixed state. Rather, the landraces from Central and South Asia revealed the highest accumulation of rare and normal alleles and should therefore be kept in high consideration for increasing diversity of modern breeding programs. Alternatively, the five clusters of ICARDA, CIMMYT, developed countries, ‘Om Rabi’ derivatives, and Italian breeding showed limited admixture with each other (**Figure 3**) and therefore their inter-hybridization is a possible source of genetic diversity. Still, this will be possible only if the exchange of seeds for breeding purposes is kept free and unobstructed.

## Author Contributions

HK, AS, FB, and RO conceived and designed the experiments; HK, AS, and MG performed the laboratory procedures; HK, AS, MG, AA-A, AA, AF-M, BB, FB, and RO analyzed the data; HK and FB wrote the paper; AS, MG, AA-A, AA, AF-M, BB, and RO edited and provided critical review of the manuscript. All authors read and approved the final manuscript.

## Conflict of Interest Statement

The authors declare that the research was conducted in the absence of any commercial or financial relationships that could be construed as a potential conflict of interest.

## Footnotes

1 www.affymetrix.com

## Supplementary Material

**FIGURE S1 |** Country of origin of the durum wheat core collection lines (landraces in black, modern lines in gray).

**FIGURE S2 |** Neighbor-joining tree based on SNPs data using Rogers’ genetic distance for 370 lines of durum wheat. Genotypes are coded based on the output of DAPC clustering, and clusters are defined using the vertical black line set at 60% similarity.

**FIGURE S3 |** Spike morphology of the two centers of origin or diversity of landraces. **(A)** Levantine landraces, **(B)** *T. abyssinicum* type.

**TABLE S1 |** Complete list with pedigree, origin, IG, and sub-population assignment for each software.
