# Supplementary figures and images for: Genetic Diversity within a Global Panel of Durum Wheat (Triticum durum) Landraces and Modern Germplasm Reveals the History of Alleles Exchange
Source: Front Plant Sci. 2017 Jul 18;8:1277. doi: 10.3389/fpls.2017.01277 (PMC5513985; doi:10.3389/fpls.2017.01277)

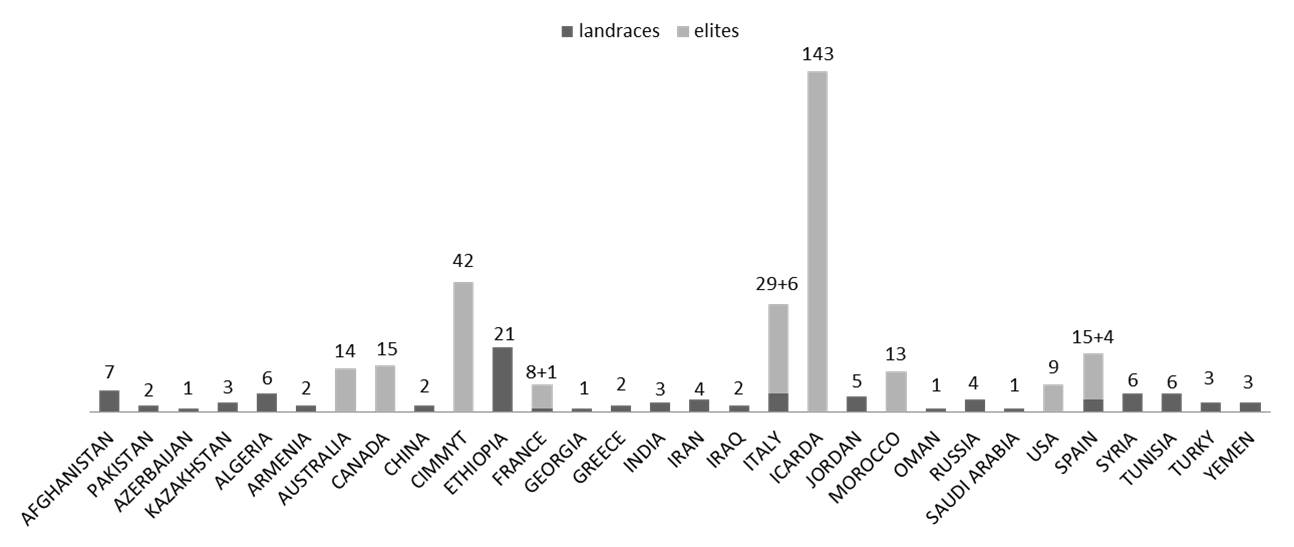

Supplement: Supplementary file 1 [file Image_1.JPEG]

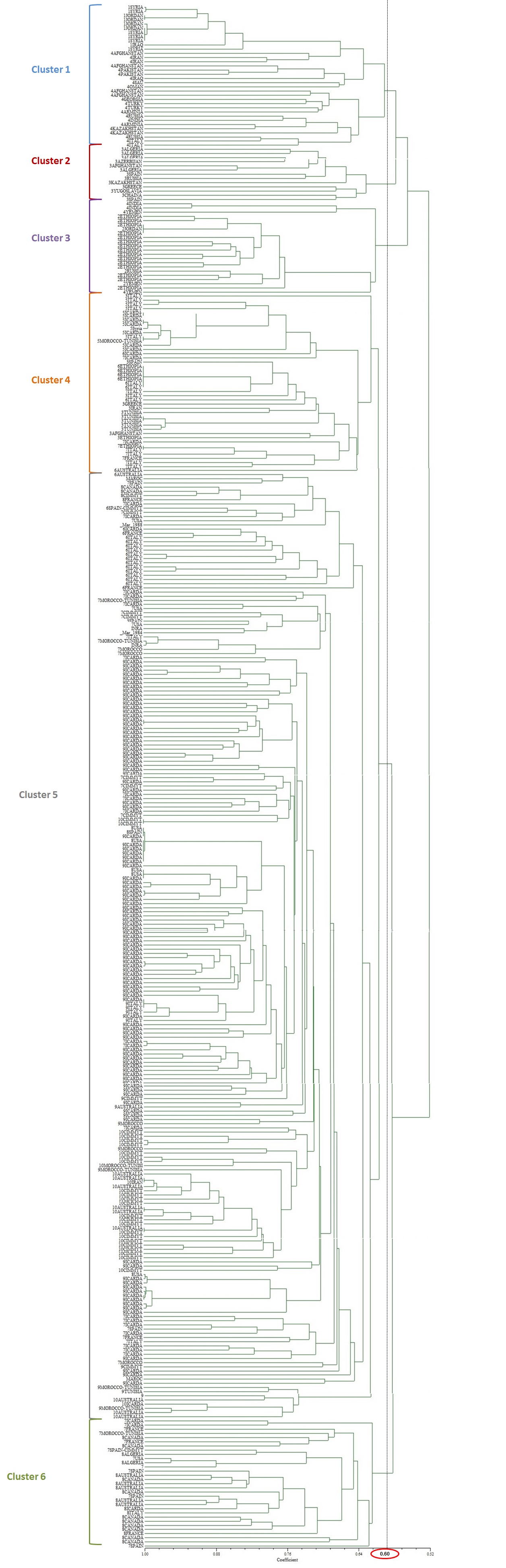

Supplement: Supplementary file 2 [file Image_2.JPEG]

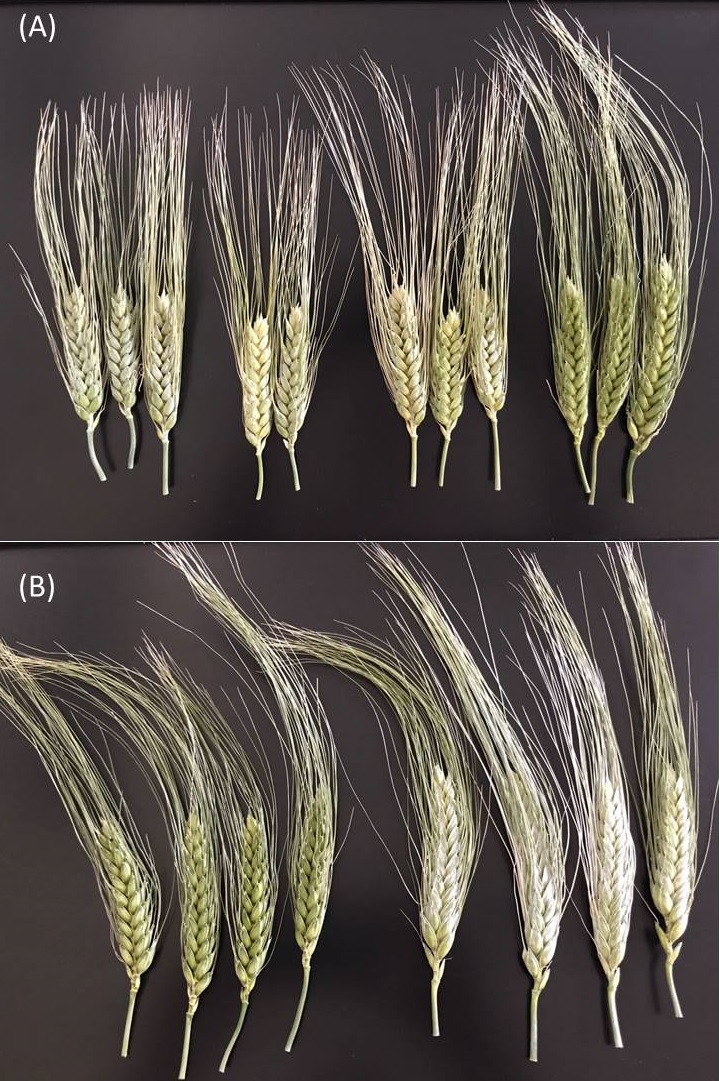

Supplement: Supplementary file 3 [file Image_3.JPEG]
